# Genome-wide identification and characterization of the MADS-box gene family in *Salix suchowensis*

**DOI:** 10.7717/peerj.8019

**Published:** 2019-11-07

**Authors:** Yanshu Qu, Changwei Bi, Bing He, Ning Ye, Tongming Yin, Li-an Xu

**Affiliations:** 1 Co-Innovation Center for Sustainable Forestry in Southern China, Nanjing Forestry University, Nanjing, China; 2 School of Biological Science and Medical Engineering, Southeast University, Nanjing, China; 3 College of Information Science and Technology, Nanjing Forestry University, Nanjing, China

**Keywords:** Gene family, MADS-box, Phylogenetic analysis, Expression, Genome-wide characterization, Willow

## Abstract

MADS-box genes encode transcription factors that participate in various plant growth and development processes, particularly floral organogenesis. To date, MADS-box genes have been reported in many species, the completion of the sequence of the willow genome provides us with the opportunity to conduct a comprehensive analysis of the willow MADS-box gene family. Here, we identified 60 willow MADS-box genes using bioinformatics-based methods and classified them into 22 M-type (11 Mα, seven Mβ and four Mγ) and 38 MIKC-type (32 MIKCc and six MIKC*) genes based on a phylogenetic analysis. Fifty-six of the 60 SsMADS genes were randomly distributed on 19 putative willow chromosomes. By combining gene structure analysis with evolutionary analysis, we found that the MIKC-type genes were more conserved and played a more important role in willow growth. Further study showed that the MIKC* type was a transition between the M-type and MIKC-type. Additionally, the number of MADS-box genes in gymnosperms was notably lower than that in angiosperms. Finally, the expression profiles of these willow MADS-box genes were analysed in five different tissues (root, stem, leave, bud and bark) and validated by RT-qPCR experiments. This study is the first genome-wide analysis of the willow MADS-box gene family, and the results establish a basis for further functional studies of willow MADS-box genes and serve as a reference for related studies of other woody plants.

## Introduction

MADS-box genes, which are an important class of transcription factors in eukaryotes, are ubiquitous in animals, plants and yeast and play significant roles in the growth and development of these organisms (Alvarez-Buylla et al., 2000; Becker & Theissen, 2003). Specifically, almost all of these genes participate in all stages of growth and development in plants, particularly the development of floral organs (Zhang et al., 2017). The name MADS-box is derived from the four first letters of *MCM1* from *Saccharomyces cerevisiae*, *AGAMOUS* from *Arabidopsis*, *DEFICIENS* from snapdragon and *SRF4* from humans, and the proteins encoded by these genes contain a highly conserved region called the MADS-box that is approximately 60 amino acid residues in length (Messenguy & Dubois, 2003).

Evolutionarily, MADS-box genes are divided into two major categories (type I and type II). Type I MADS-box genes are further divided into Mα, Mβ and Mγ. Type II genes, which also known as the MIKC type due to their common structure of four domains, can be further divided into two subtypes (MIKCc and MIKC*) based on different structural features (Henschel et al., 2002; Kwantes, Liebsch & Verelst, 2012; Parenicova et al., 2003). Additionally, another method exists for MADS-box gene classification. For example, when the *Arabidopsis* gene family was classified, a Bayesian method was used to divide the genes into five subclasses (Mα, Mβ, Mγ, M*δ* and MIKC) (Parenicova et al., 2003). Structurally, almost all MADS-box genes contain a conserved MADS domain consisting of 60 amino acid residues at the N- terminus, and this domain is responsible for binding the CArG-box (CC(A/T)_6_GG) in the regulatory region of target genes (Messenguy & Dubois, 2003).

The main difference between plant type I and type II MADS-box genes is whether they contain a K domain. Type I MADS-box genes contain only one highly conserved MADS domain with no or few introns, and their abundance is lower at the transcriptional level. Type II MADS-box genes have a multi-intron structure with the exception of the highly conserved MADS domain. In order from the N- to the C-terminus, this gene type also contains the intervening (I) domain, keratin (K) domain, and C-terminal (C) region (De Bodt et al., 2003; Smaczniak et al., 2012a). The I domain is a non-conserved region composed of 31–35 amino acid residues that assists with the binding to form dimers and complexes with DNA. The K domain is the second conserved region following the MADS domain and is a coiled coil with a length of approximately 70 amino acid residues. This domain is a structural unit responsible for dimerization and is also considered a characteristic sequence of MADS-box transcription factors in plants (K domains only exist in plants) (Wu et al., 2006). The C-terminal region is the most variable region and has been validated to play an important role in the formation and transcriptional activation of protein complexes.

Previous studies have shown that MIKCc-type genes play a more important role in plant floral organ development (Grimplet, Martinez-Zapater & Carmona, 2016; Weigel & Meyerowitz, 1994). At first, MIKCc-type gens were considered as floral organ identity genes in *Antirrhinum majus* and *Arabidopsis thaliana*. Further molecular and genetic analysis subdivided these genes into five different classes (A, B, C, D and E), to specify the identity of sepals (A), petals (A + B + E), stamens (B + C + E), carpels (C + E) and ovules (D) (Grimplet, Martinez-Zapater & Carmona, 2016; Theissen, 2001; Weigel & Meyerowitz, 1994; Xu et al., 2014). The genes belonging to the above five functional categories in *Arabidopsis* include: *APETALA1* (*AP1*) in class A, *PISTILATA* (*PI*) and *APETALA3* (*AP3*) in class B, *AGAMOUS* (*AG*) in class C, *SEEDSTICK/ AGAMOUS*-LIKE 1 (*STK/AGL11*) and *SHATTERPROOF* (*SHP*) in class D and *SEPALLATA* (*SEP1*, *SEP2*, *SEP3*, *SEP4*) genes in class E (Theissen, 2001; Theissen, Rumpler & Gramzow, 2018). In addition, MIKCc genes in the *AG* and *APETALA1*/*FRUITFULL* (*AP1*/*FUL*) subclasses are also involved in the development of fruit and seed. *FLOWERING LOCUS C* (*FLC*), *SUPRESSOR OF OVEREXPRESSION OF CONSTANTS 1* (*SOC1*) and *SHORT VEGETATIVE PHASE* (*SVP*) are participate in different regulatory networks controlling flowering time and flower initiation (Immink et al., 2003; Li et al., 2008; Smaczniak et al., 2012b). In view of the important role of the MADS-box gene family in the plant lifecycle, researchers have identified this gene family in a variety of plants, including *Arabidopsis thaliana*, *Oryza sativa*, *Brachypodium distachyon*, *Malus domestica*, *Ziziphus jujuba*, and *Populus trichocarpa* (Arora et al., 2007; Bi et al., 2016; Kaufmann, Melzer & Theissen, 2005; Leseberg et al., 2006; Ng & Yanofsky, 2001; Parenicova et al., 2003; Tian et al., 2015; Wei et al., 2014; Zhang et al., 2017). *Salix suchowensis* is a general term for the type of woody plants belonging to the genus *Salix*, which include deciduous shrubs and arbors with a long cultivation history in China. Because of their strong adaptability to the environment and short generation period, willows have been widely recognized as an important renewable source of bioenergy that can be used in cogeneration to meet today’s rapidly increasing demand for renewable resources. In addition, willows have good economic value; for example, they can be used to make boxes and process antirheumatic Chinese medicinal herbs and are cultivated as ornamental trees (Bi et al., 2016; Kuzovkina & Quigley, 2005). However, the MADS-box gene family in willows has not been identified. After the draft of the *Salix suchowensis* genome sequence was completed in 2014, approximately 96% of the genetic loci were effectively annotated, and transcriptome data became easily available (Dai et al., 2014). Therefore, we have the opportunity to identify the MADS-box gene family from the willow whole-genome protein data.

Based on the latest published *Salix suchowensis* genome database, we identified members of the MADS-box gene family and analyzed their chromosomal locations, exon-intron structures, evolution and gene expression profiles. These results establish a basis for further functional studies of willow MADS-box genes and serve as a reference for related studies of other woody plants.

## Materials and Methods

### Datasets and sequence retrieval

All the latest version files related to the *Salix suchowensis* genome sequence that were used for the identification of MADS-box genes were downloaded from the website of the Bioinformatics Laboratory of the Information College of Nanjing Forestry University (https://figshare.com/articles/Willow_gene_family/9878582/1). *Arabidopsis* genomic data and 89 MADS-box sequences were downloaded from The Arabidopsis Information Resource (TAIR, http://www.arabidopsis.org/index.jsp) with the accession numbers reported by Parenicová et al., and the MADS-box protein data for rice were obtained from the Rice Genome Annotation Project (RGAP, http://rice.plantbiology.msu.edu/index.shtml) (Kawahara et al., 2013; Parenicova et al., 2003).

### Identification and distribution of MADS-box genes in willows

The method used to identify proteins corresponding to the willow MADS-box genes was similar to that used for other species (Duan et al., 2015; Tian et al., 2015; Wei et al., 2014). Fasta and Stockholm format files for the MADS-box domains were retrieved from the Pfam database (release 31.0, http://pfam.xfam.org/) with the accession number ‘PF00319’ (Finn et al., 2016). To obtain potential proteins, an alignment of MADS-box seed sequences in the Stockholm format was generated by a tool in the HMMER programs (hmmbuild) to build an HMM model, and then the model was used to search all willow proteins using another tool (hmmsearch) with the default parameters (Eddy, 1998). BLASTp ( *E*-value = 1^−3^) was used to align the Fasta profile downloaded from the PFAM website with all willow protein sequences (Willow.gene.pep) (Camacho et al., 2009). The potential willow MADS-box genes were obtained by taking the intersection of the above two results. To validate the confidence of these genes, we used the SMART programme (http://smart.embl-heidelberg.de/) to confirm whether a MADS-box domain was contained in each candidate MADS-box protein (Letunic, Doerks & Bork, 2015). Genes that did not contain an entire MADS domain were removed to identify eligible MADS-box gene family members. In addition, we used the ExPasy tool (https://www.expasy.org/tools/) to calculate the lengths, molecular weights, and isoelectric points of these putative MADS-box proteins. Finally, all identified MADS-box genes were mapped onto willow chromosomes with an in-house Perl script (http://bio.njfu.edu.cn/willow_chromosome/BuildGff3_Chr.pl). The distribution of each MADS-box gene on the willow chromosomes was plotted using the MapInspect software (https://github.com/quyanshu/Willow-gene-family/blob/master/BuildGff3_Chr.pl), and these genes were renamed based on their chromosomal distributions.

### Multiple alignment and phylogenetic analysis of the willow MADS-box genes

The sequence logo of the identified willow MADS-box genes was generated using the web-based application WebLogo3 (http://weblogo.threeplusone.com) with the default parameters (Crooks et al., 2004). To obtain the conserved MADS-box domains of these willow MADS-box genes, we employed the online tool SMART and the PFAM database and used ClustalX (version 2.1) to perform multi-sequence alignment of the MADS-box domains obtained from SMART (Larkin et al., 2007). The online tool BoxShade (http://www.ch.embnet.org/software/BOX_form.html) was then used to colour the resulting alignment.

In general, all Willow MADS genes can be divided into two categories (M-type and MIKC-type) through the PlantTFDB website (http://planttfdb.cbi.pku.edu.cn/). However, to obtain a better subgroup classification of these genes, a multiple sequence alignment including willow (SsMADS) and *Arabidopsis* (AtMADS) MADS-box proteins was performed using Muscle, and a NJ tree was built with MEGA 7.0 based on this alignment (Edgar, 2004; Jin et al., 2014; Kumar, Stecher & Tamura, 2016). A NJ tree was then established for all *Arabidopsis* MADS-box proteins to check the reliability of this method (Duan et al., 2015). A phylogenetic tree was constructed using a similar method with the identified SsMADS domains and 66 rice MADS-box core domains (OsMADS). Additionally, a phylogenetic tree was built based on the identified SsMADS proteins.

Subsequently, to enable better comparison of MADS-box genes in Salicaceae, a phylogenetic tree was established for all SsMADS and *Populus trichocarpa* MADS-box genes. The method was consistent with that described above.

Finally, the orthologues of each SsMADS gene in *A. thaliana*, rice and *Populus* were determined based on the phylogenetic trees of the MADS-box domains or proteins and the BLASTP programme results (bi-direction, best hit, *E*-value = 1e^−20^) (Chen et al., 2007).

### Gene structure analysis of the willow MADS-box genes

The intron-exon structures of the willow MADS-box genes were contained in our own assembled protein annotation file. After annotation information for all SsMADS genes was extracted using a Perl language script, an intron-exon structure diagram was obtained from the online tool GSDS (Gene Structure Display server, http://gsds.cbi.pku.edu.cn/) (Hu et al., 2015).

Multi-sequence and BLASTp alignments (*E*-value = 1e−20) were performed to obtain the similarities between these SsMADS genes. To estimate gene duplication events in the SsMADS genes, the following metrics were set: (1) the proportion of regions used for alignment of the longer gene should exceed 65% and (2) the similarity of the aligned regions should exceed 65% (Bi et al., 2016).

To better reveal the structural features of the SsMADS proteins, the online tool Multiple Expectation Maximization for Motif Elicitation (MEME, http://meme-suite.org/) was used to predict conserved motifs in the encoded SsMADS proteins (Bailey et al., 2006). The parameters were set to a repeat motif site of any number, a maximum number of motifs of 15, and a width of each motif ranging from 6 to 60 residues. The web-based software 2ZIP (http://2zip.molgen.mpg.de/) was used to verify whether these SsMADS proteins contained the Leu zipper motif, and other important conserved motifs, including LXXLL and LXLXLX, were searched manually (Bornberg-Bauer, Rivals & Vingron, 1998).

### Expression analysis of the willow MADS-box genes

To obtain more information regarding the roles of MADS-box genes in willows, RNA-Seq data from the sequenced genotype were used to quantify the expression levels of MADS-box genes in five tissues from *S. suchowensis*. The BWA programme was used to map back the *S. suchowensis* RNA-Seq reads from five tissues (roots, stems, leaves, buds and skins) onto the SsMADS gene sequences, and the number of mapped reads for each SsMADS gene in RPKM (reads per kilo base per million mapped reads) was calculated manually and standardized using Log_2_ RPKM (Li & Durbin, 2009; Wagner, Kin & Lynch, 2012). A gene expression profile heat map was drawn with Bioconductor (pheatmap package) (Gentleman et al., 2004).

### RNA isolation and Real-time quantitative RT-qPCR

Total RNA was isolated from five frozen willow tissues using an RNA kit (RNAprep Pure Plant Kit, Tiangen, Beijing, China), the specific procedures can be found in the manufacturer’s instructions. The quality and concentration of different RNA samples were determined by a NanoDrop 2000 c spectrophotometer (Thermo Scientific, Wilmington, DE, USA) and 1.0 percent (w/v) agarose gel electrophoresis. cDNA was synthesized from 1,000 ng of total RNA in a 20 µL reaction volume using PrimeScript™ RT Master Mix (TaKaRa, Dalian, China) according to the manufacturer’s instructions. The resulting cDNA was then diluted three-fold and stored at −20 °C for the subsequent RT-qPCR assays.

For gene expression quantification, using the Oligo 7 algorithm (https://en.freedownloadmanager.org/Windows-PC/OLIGO.html) to design specific primers for each *SsMADS* gene, primer details are listed in the Table S1. The expression of 6 *SsMADS* genes was verified using RT-qPCR with a total volume of 10 µL per reaction (1 µL of cDNA, 1 µL of the forward and reverse primers, 5 µL of SYBR Green mix (TaKaRa, Dalian, China) and 3 µL ddH_2_O) and performed on a StepOnePlus™ System (Applied Biosystems). The reactions were performed under the following conditions: 95 °C for 30 s and 50 cycles of 95 °C for 15 s and 60 °C for 1 min. The specificity of the amplicon for each primer pair was verified by melting curve analysis. All experiments were performed in three biological replicates; each replicate being measured in triplicate. Relative expression levels were calculated using the 2^−ΔΔ*Ct*^ method, with the OTU-like cysteine protease gene (*OTU*) of *S. suchowensis* as reference gene.

## Results

### Identification and characterization of the MADS-box gene family in *S. suchowensis*

Sixty-four MADS-box genes were obtained using the HMMER toolkit to search the Hidden Markov Model of the MADS-box DNA-binding domain in the willow whole-genome protein sequence. The accuracy of the results was verified through BLASTP and HMMER mutual verification. Subsequently, the potential MADS-box genes were submitted to the SMART website for further verification. Four genes were removed due to lack of a MADS domain, and the remaining 60 probable MADS-box genes were selected as MADS-box superfamily members.

To better understand the MADS domain of *S. suchowensis*, a sequence logo and a multiple alignment with 60 SsMADS domains were generated. Amino acids 3, 23, 24, 27, 30, 31, and 34 were highly conserved, which confirmed conservation of the MADS domain (Fig. S1).

**Figure 1:**
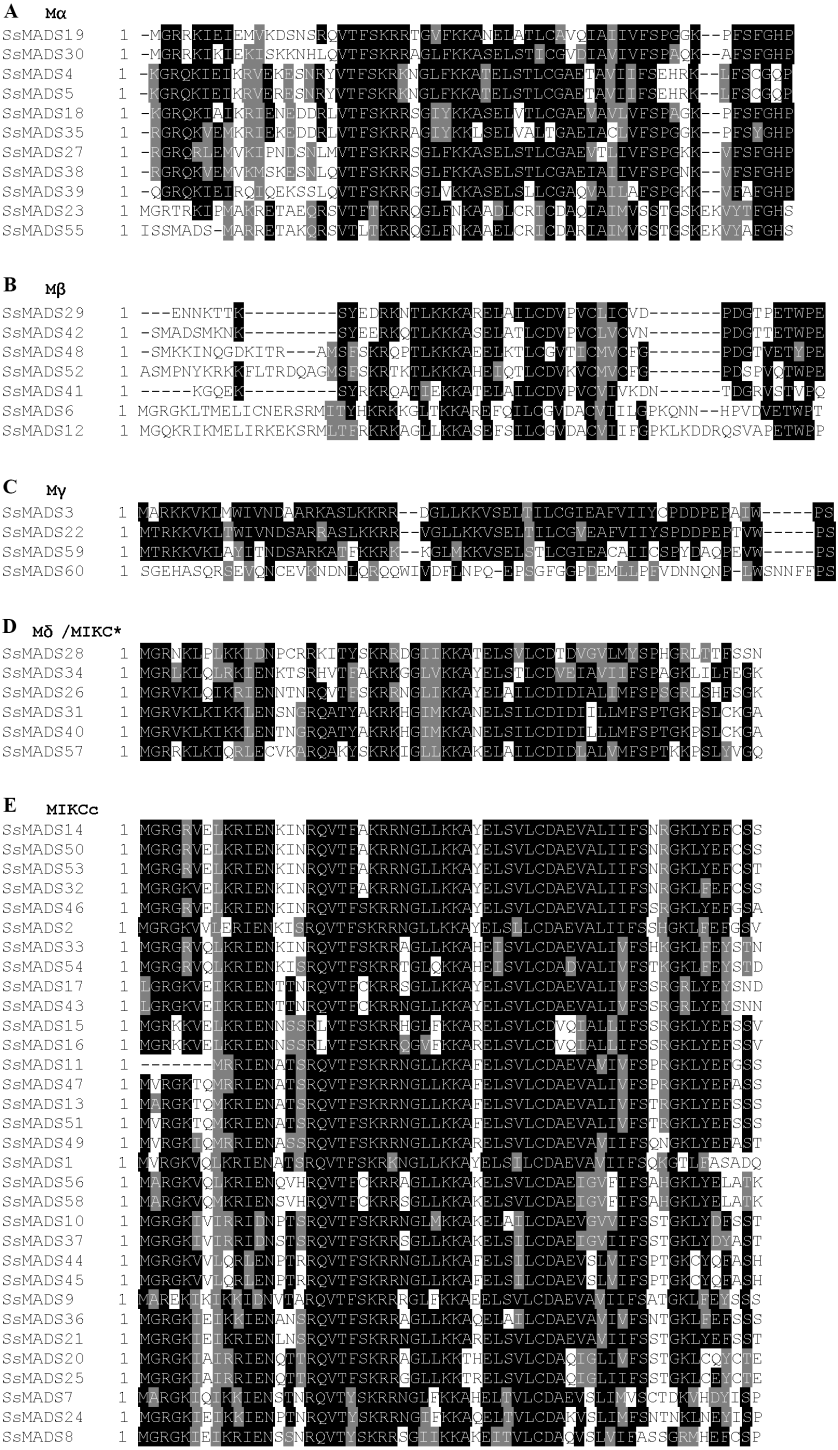
Comparison of the MADS-box domains from the 60 willow MADS-box genes. The multi-alignment was performed using the ClustalX programme (version 2.1) and coloured using the online tool BoxShade (http://www.ch.embnet.org/software/BOX_form.html). Black indicates a highly conserved region. (A) Mα subgroup. (B) Mβ subgroup. (C) Mγ subgroup. (D) M*δ*/MIKC* subgroup. (E) MIKCc subgroup.

As shown in Fig. 1, the structures of the type I and type II SsMADS genes were quite different, and the type II SsMADS genes were more conserved than the type I genes. The MIKCc subgroup was the most conserved type, and several conserved motifs, including RQVT and RIEN, were concentrated at the N-terminus. The similarities between types I and II mainly occurred in the central region near the C-terminus. For example, differences in the N-terminal amino acids in *Physcomitrella patens* were reported to determine the differences between type I and type II MADS-box genes, whereas MIKCc and MIKC* are distinguished by the C-terminus (Henschel et al., 2002). In general, the type II MADS-box genes of *S. suchowensis*, particularly the MIKCc subgroup, were more conserved.

Detailed characteristics, including the classification, chromosomal distribution, homologous genes, and related physicochemical properties, of the SsMADS genes are listed in Table 1. As shown in Table 1, these protein sequences ranged from 80 amino acids (*SsMADS34*) to 894 amino acids (*SsMADS40*), with an average of 277 amino acids. Furthermore, the range of isoelectric points (PIs) also showed a large fluctuation, from 4.44 (*SsMADS23*) to 10.33 (*SsMADS34*), and the molecular weights (MWs) ranged from 9.20 kDa (*SsMADS34*) to 98.51 kDa (*SsMADS40*). These findings reflect the high complexity of willow MADS-box genes.

**Table 1:** Detailed information for the MADS-box gene family in willow.

Gene	Sequence ID	Class	Chr	Orthologue			Physicochemical characteristics			Introns
				PtMADS	AtMADS	OsMADS	Length (aa)	MW (kDa)	PI	
*SsMADS1*	willow_GLEAN_10012476	MIKCc	chr01	101	20	50	209	24.00	8.53	6
*SsMADS2*	willow_GLEAN_10012473	MIKCc	chr01	97	6	6,17	232	27.06	9.1	7
*SsMADS3*	willow_GLEAN_10014137	Mγ	chr01	46	–	–	401	43.52	7.05	0
*SsMADS4*	willow_GLEAN_10007397	Mα	chr01	47,48	–	–	530	60.23	6.67	8
*SsMADS5*	willow_GLEAN_10007399	Mα	chr01	47,48	–	–	212	24.34	6.22	0
*SsMADS6*	willow_GLEAN_10011253	Mβ	chr01	90	–	–	595	67.62	9.16	7
*SsMADS7*	willow_GLEAN_10022499	MIKCc	chr02	69	APETALA3	16	220	25.64	9.15	6
*SsMADS8*	willow_GLEAN_10020801	MIKCc	chr02	64	PISTILLATA	16	829	92.12	6.57	7
*SsMADS9*	willow_GLEAN_10020993	MIKCc	chr02	68	24	47	222	24.91	8.33	6
*SsMADS10*	willow_GLEAN_10021024	MIKCc	chr02	66	16	57	217	24.78	9.59	5
*SsMADS11*	willow_GLEAN_10011768	MIKCc	chr02	71	14	50	165	18.94	9.35	4
*SsMADS12*	willow_GLEAN_10020216	Mβ	chr02	67,102	–	–	405	45.69	7.57	0
*SsMADS13*	willow_GLEAN_10025520	MIKCc	chr03	94	14	50	287	32.58	10.07	5
*SsMADS14*	willow_GLEAN_10008017	MIKCc	chr03	95	2,9	7∕45, 8∕24	245	27.96	8.58	7
*SsMADS15*	willow_GLEAN_10008015	MIKCc	chr03	35,26	–	6,17	263	29.40	9.31	4
*SsMADS16*	willow_GLEAN_10008014	MIKCc	chr03	35,26	–	6,17	218	24.65	7.83	6
*SsMADS17*	willow_GLEAN_10017246	MIKCc	chr04	25	AGAMOUS	58	350	39.19	9.3	8
*SsMADS18*	willow_GLEAN_10011967	Mα	chr04	21	–	–	194	21.74	9.08	0
*SsMADS19*	willow_GLEAN_10011966	Mα	chr04	27	29	–	178	20.10	9.96	0
*SsMADS20*	willow_GLEAN_10009082	MIKCc	chr05	53	–	29	219	25.34	8.54	4
*SsMADS21*	willow_GLEAN_10027002	MIKCc	chr06	43	15	57	250	28.08	8.65	7
*SsMADS22*	willow_GLEAN_10025994	Mγ	chr06	44	48	–	469	51.05	5.84	0
*SsMADS23*	willow_GLEAN_10026418	Mα	chr06	12,42	–	–	374	40.67	4.44	0
*SsMADS24*	willow_GLEAN_10012682	MIKCc	chr07	49	APETALA3	16	229	26.62	8.84	6
*SsMADS25*	willow_GLEAN_10007501	MIKCc	chr07	53	90	29	233	27.19	7.71	5
*SsMADS26*	willow_GLEAN_10007031	MIKC*	chr07	52	104	63	364	41.19	5.61	10
*SsMADS27*	willow_GLEAN_10014009	Mα	chr07	6	43	–	254	28.19	9.17	1
*SsMADS28*	willow_GLEAN_10014039	MIKC*	chr07	51	–	–	169	19.01	9.3	4
*SsMADS29*	willow_GLEAN_10024615	Mβ	chr08	84	–	–	202	22.90	6	0
*SsMADS30*	willow_GLEAN_10024753	Mα	chr08	17	–	–	197	22.70	9.36	0
*SsMADS31*	willow_GLEAN_10025082	MIKC*	chr08	85	30	68	357	39.79	6.95	9
*SsMADS32*	willow_GLEAN_10025158	MIKCc	chr08	87,95	2,9	7∕45, 8∕24	241	27.62	5.65	7
*SsMADS33*	willow_GLEAN_10025159	MIKCc	chr08	86	7	15	212	24.53	8.48	5
*SsMADS34*	willow_GLEAN_10008129	MIKC*	chr09	57	–	–	80	9.23	10.33	1
*SsMADS35*	willow_GLEAN_10022978	Mα	chr09	19	–	–	205	23.07	5.29	0
*SsMADS36*	willow_GLEAN_10023049	MIKCc	chr09	15	15	29	259	29.39	8.81	7
*SsMADS37*	willow_GLEAN_10024397	MIKCc	chr09	89,66	44	57,61	263	30.14	9.39	6
*SsMADS38*	willow_GLEAN_10024365	Mα	chr09	18	43	–	416	46.75	9.62	2
*SsMADS39*	willow_GLEAN_10021705	Mα	chr10	29,7	–	–	203	23.09	5.25	0
*SsMADS40*	willow_GLEAN_10013611	MIKC*	chr10	85	30	68	894	98.51	6.62	13
*SsMADS41*	willow_GLEAN_10019310	Mβ	chr10	2	–	–	342	37.50	8.32	1
*SsMADS42*	willow_GLEAN_10004380	Mβ	chr10	1	–	–	201	22.46	5.02	0
*SsMADS43*	willow_GLEAN_10005930	MIKCc	chr11	41	AGAMOUS	3	227	25.81	9.62	5
*SsMADS44*	willow_GLEAN_10013792	MIKCc	chr12	103	–	34	135	15.72	9.47	3
*SsMADS45*	willow_GLEAN_10006110	MIKCc	chr13	103	–	34	232	26.73	8.84	5
*SsMADS46*	willow_GLEAN_10016051	MIKCc	chr14	82	6	7,16	218	25.40	9.85	6
*SsMADS47*	willow_GLEAN_10016052	MIKCc	chr14	83	20	50	218	25.38	9.55	6
*SsMADS48*	willow_GLEAN_10004716	Mβ	chr15	60	–	–	220	25.26	6.85	0
*SsMADS49*	willow_GLEAN_10009701	MIKCc	chr15	–	20	50	266	31.05	8.98	7
*SsMADS50*	willow_GLEAN_10023443	MIKCc	chr16	95	2,9	7∕45, 8∕24	267	30.54	6.26	8
*SsMADS51*	willow_GLEAN_10003749	MIKCc	chr16	94	14	50	255	28.99	9.34	7
*SsMADS52*	willow_GLEAN_10002958	Mβ	chr16	20	–	–	265	30.53	5.37	0
*SsMADS53*	willow_GLEAN_10003926	MIKCc	chr17	23	29	7∕45, 8∕24	245	28.17	8.27	7
*SsMADS54*	willow_GLEAN_10003927	MIKCc	chr17	14,26	8	14,15	238	27.54	9.18	6
*SsMADS55*	willow_GLEAN_10006611	Mα	chr18	–	–	–	310	33.64	4.74	0
*SsMADS56*	willow_GLEAN_10013302	MIKCc	chr19	72,31	12	26	321	36.31	8.47	4
*SsMADS57*	willow_GLEAN_10001835	MIKC*	N/A	45	–	–	82	9.51	9.9	1
*SsMADS58*	willow_GLEAN_10001302	MIKCc	N/A	31	12	26	156	17.88	9.1	3
*SsMADS59*	willow_GLEAN_10001292	Mγ	N/A	34	80	–	235	26.81	9.27	0
*SsMADS60*	willow_GLEAN_10000968	Mγ	N/A	–	–	–	158	18.14	5.99	0

**Notes.**

Chr chromosome numbers N/A not available – not detected

### Chromosome distribution characteristics of the willow MADS-box genes

**Figure 2:**
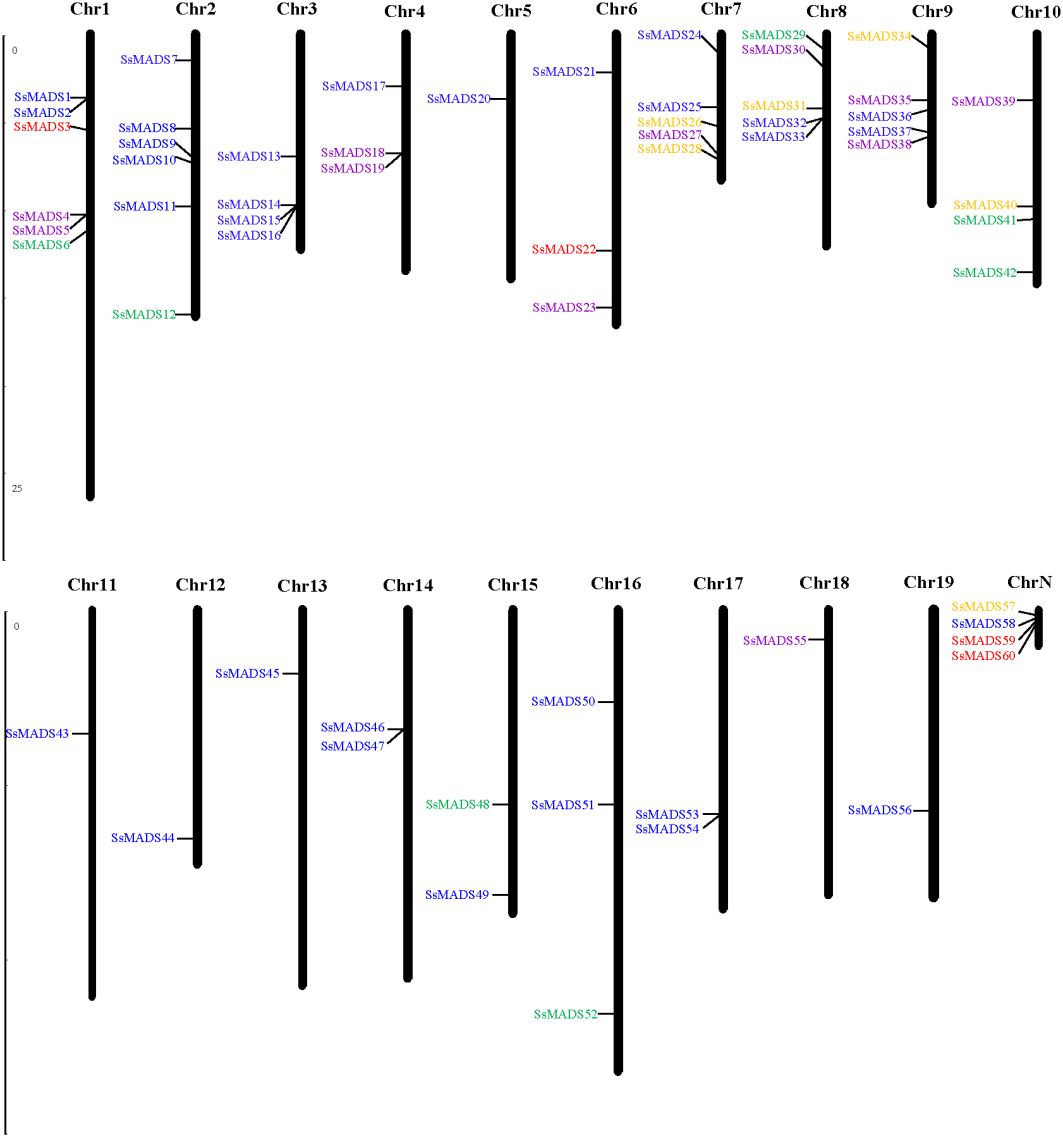
Chromosomal localization of the 60 willow MADS-box genes. The number of each chromosome is given above the lines. The left side of each chromosome is related to the approximate physical location of each MADS-box gene. The four unmapped genes are shown on ChrN. Purple indicates Mα, green indicates Mβ, brown indicates Mγ, yellow indicates MIKC*, and blue indicates MIKCc.

Fifty-six of the 60 SsMADS genes were distributed on 19 putative willow chromosomes, and these genes were renamed *SsMADS1* to *SsMADS56* based on their locations on the chromosomes. Only four SsMADS genes (*willow_GLEAN_10001835*, *willow_GLEAN_10001302*, *willow_GLEAN_10001292*, and *willow_GLEAN_10000968*) could not be mapped onto any chromosome, and these were renamed *SsMADS57*, *SsMADS58*, *SsMADS59*, and *SsMADS60*, respectively. As demonstrated in Fig. 2, chromosomes (Chr) 1 and 2 contained the largest number of SsMADS genes (six genes per chromosome), followed by Chr7, Chr8 and Chr9 (five genes per chromosome). Four SsMADS genes were found on Chr3 and Chr10, and three were found on Chr4, Chr6 and Chr16. Additionally, three chromosomes (Chr14, Chr15, and Chr17) contained two SsMADS genes, whereas only one SsMADS gene was found on Chr5, Chr11, Chr12, Chr13, Chr18 and Chr19. ChrN indicated that genes were not mapped on any chromosome.

The distribution of the MADS-box genes was not random; instead, an enrichment region showed a relatively high density on some chromosomes or chromosome fragments. Previous studies showed that a single chromosome region within 200 kb that contained two or more genes could be defined as a gene cluster (He et al., 2012; Holub, 2001). Genes that are used in large amounts are clustered in the genome to facilitate the rapid synthesis of large numbers of transcripts, which is important for predicting the potential function of co-expressed or clustered genes in angiosperms. According to the present study, a total of 21 SsMADS genes in willows were clustered into 11 clusters and distributed on nine chromosomes (Fig. 2). Two gene clusters were found on Chr1, including four SsMADS genes; one gene cluster each was distributed on Chr2, Chr3, Chr4, Chr7, Chr8, Chr9, Chr14 and Chr17. Three SsMADS genes were distributed in the gene cluster on Chr3, whereas no gene cluster was found on the other ten chromosomes.

### Classification of MADS-box genes in willows

**Figure 3:**
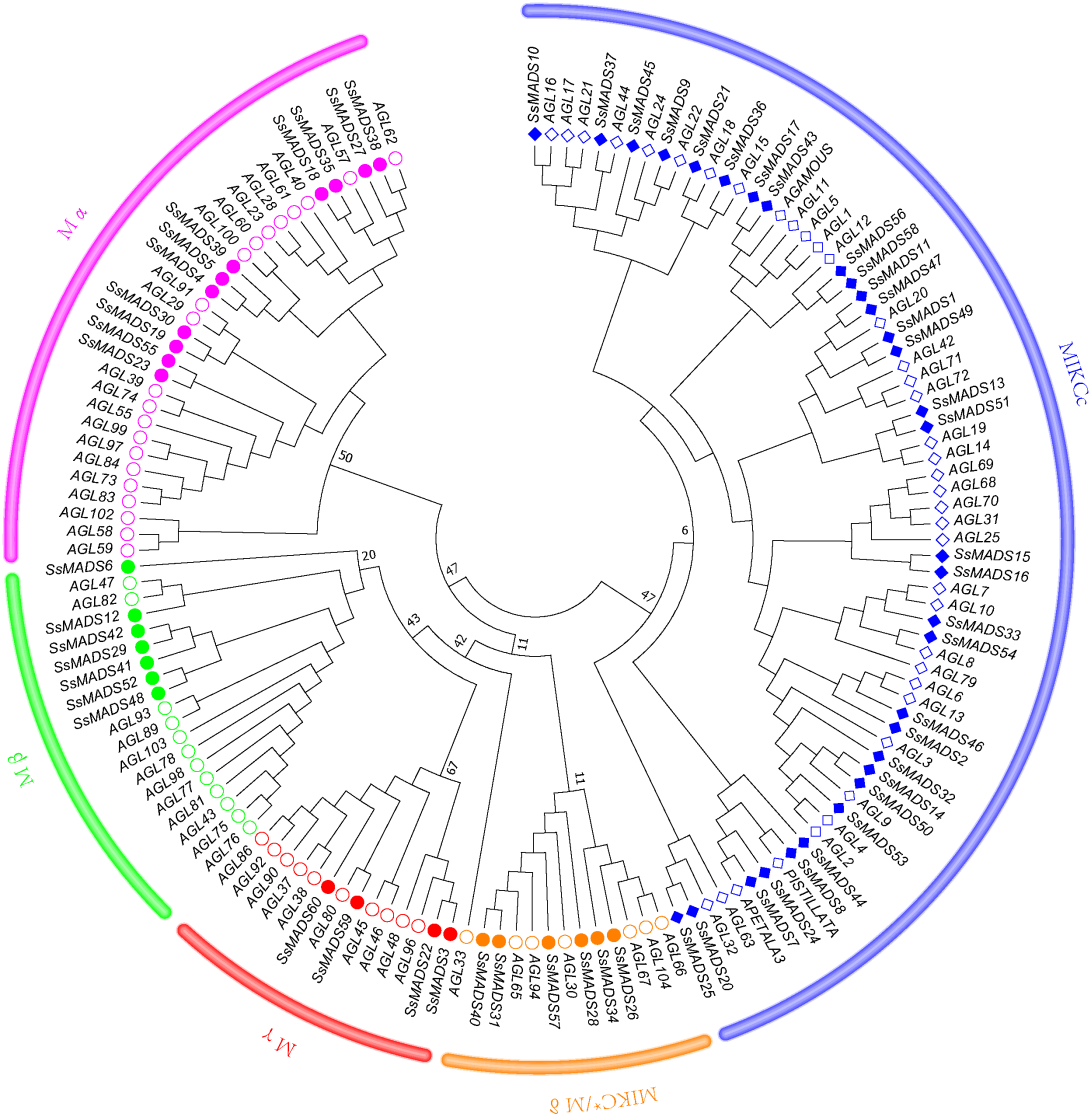
Phylogenetic tree of *S. suchowensis* and *A. thaliana* MADS-box proteins. A total of 60 MADS-box proteins from *S. suchowensis* and 88 from *A. thaliana* were used to construct a NJ tree using MEGA 7. Different shapes and colours represent different species and gene categories.

To better classify these SsMADS genes, a phylogenetic tree (NJ tree) was constructed using 88 AtMADS proteins from *A. thaliana* and the 60 SsMADS proteins identified in the present study. Based on the phylogenetic tree and structural features of the MADS-box proteins, all 60 SsMADS genes could be divided into two main groups (type I and type II) (Fig. 3). A total of 22 members were classified as type I (M-type), and these were further classified into Mα, Mβ and Mγ, with 11, seven and four members each, respectively. The remaining 38 members were categorized as type II (MIKC-type), which included 32 MIKCc-type and six MIKC*-type members. Furthermore, a similar classification was obtained with the NJ tree established for the 60 SsMADS domains and 66 rice MADS domains (Fig. S2). To better investigate the role of MADS-box genes in Salicaceae, we constructed a phylogenetic tree using 103 poplar and 60 willow MADS domains (Fig. S3). Based on the NJ tree described above, we found that most of the MADS-box genes from willows and poplars were clustered into sister pairs (40 SsMADS genes, accounting for 66.7% of all willow MADS-box genes, such as *SsMADS32-PtMADS12* and *SsMADS37-PtMADS89*) because they originated from a common ancestor.

In addition, we compared the number of willow MADS-box genes with those of the ancient tree species *Ginkgo biloba*. The *G. biloba* MADS-box genes were predicted using the same method used to predict the willow MADS-box genes. The results revealed that *G. biloba* contained only 26 MADS-box genes, which was quite different from the number found in the willow genome. The number of MADS-box gene family members of gymnosperms, such as the *Pinus taeda*, an angiosperm variety, as well as monocotyledonous plants, such as *Zea mays* and *Oryza sativa*, and dicotyledons, such as *Malus domestica* and *Glycine max*, were also analyzed (Table 2). The gymnosperm genome was larger, but the number of this gene family was much smaller than that of the angiosperms.

**Table 2:** Number of MADS-box genes in different species.

Phylum	Class	Order	Family	Species	Genome size	Total	Type I	Type II
Angiosperms	Eudicots	Malpighiales	Salicaceae	*Salix Suchowensis*	425 Mb	60	22	38
				*Populus trichocarpa*	480 Mb	103	41	64
		Rosales	Rosaceae	*Malus domestica*	742 Mb	146	64	82
		Fabales	Fabaceae	*Glycine max*	1,100 Mb	106	34	72
	Monocots	Poales	Poaceae	*Zea mays*	2,300 Mb	75	32	43
				*Oryza sativa*	466 Mb	75	28	47
				*Brachypodium distachyon*	260 Mb	57	18	39
Gymnosperm	Ginkgoopsida	Ginkgoales	Ginkgoaceae	*Ginkgo biloba*	10.61 Gb	26	/	/
	Pinopsida	Pinales	Pinaceae	*Pinus taeda*	22 Gb	11	/	/
				*Picea sitchensis*	/	17	1	16
	Cycadopsida	Cycadales	Cycadaceae	*Cycas elongata*	/	12	2	12

**Notes.**

/ not available

### Orthologues of SsMADS genes in Arabidopsis, rice and poplars

In this study, orthologous SsMADS genes in *A. thaliana*, rice and poplar were identified through a phylogenetic analysis combined with a BLAST-based method (bi-direction best hit). Finally, 35 pairs of orthologous genes from willow and *A. thaliana*, 35 pairs from willow and rice, and 57 pairs from willow and poplar were identified. The 22 type I SsMADS genes had 20 pairs of orthologous genes in poplar and five in *A. thaliana*, whereas rice contained no orthologues of the 22 type I SsMADS genes. The 38 type II SsMADS genes had 37, 30 and 35 pairs of orthologous genes in poplar, *A. thaliana*, and rice, respectively. In addition, 12 SsMADS genes were found to have identical domains in poplars (*SsMADS9*, *SsMADS14*, *SsMADS17*, *SsMADS23*, *SsMADS24*, *SsMADS26*, *SsMADS43*, *SsMADS46*, *SsMADS50*, *SsMADS51*, *SsMADS53* and *SsMADS58*), and these accounted for 20% of the total number of genes. Among these 12 genes, 11 were MIKC-type, and only *SsMADS23* was Mα; in addition, all 11 MIKC genes were found to have orthologous genes with high similarity in *Arabidopsis* and rice. For example, the similarity between *SsMADS14* and *OsMADS7/45* was 98.33%, the similarity between *SsMADS14* and *AGL2/AGL9* was 100%, the similarity between *SsMADS43* and *AGAMOUS* was 98.31%, and the similarity between *SsMADS50* and *AGL2/AGL9* was 100%.

We also found that the vast majority of SsMADS genes that did not have orthologous genes in *Arabidopsis* also had no orthologous genes in rice.

### Exon-intron structures of the SsMADS genes

**Figure 4:**
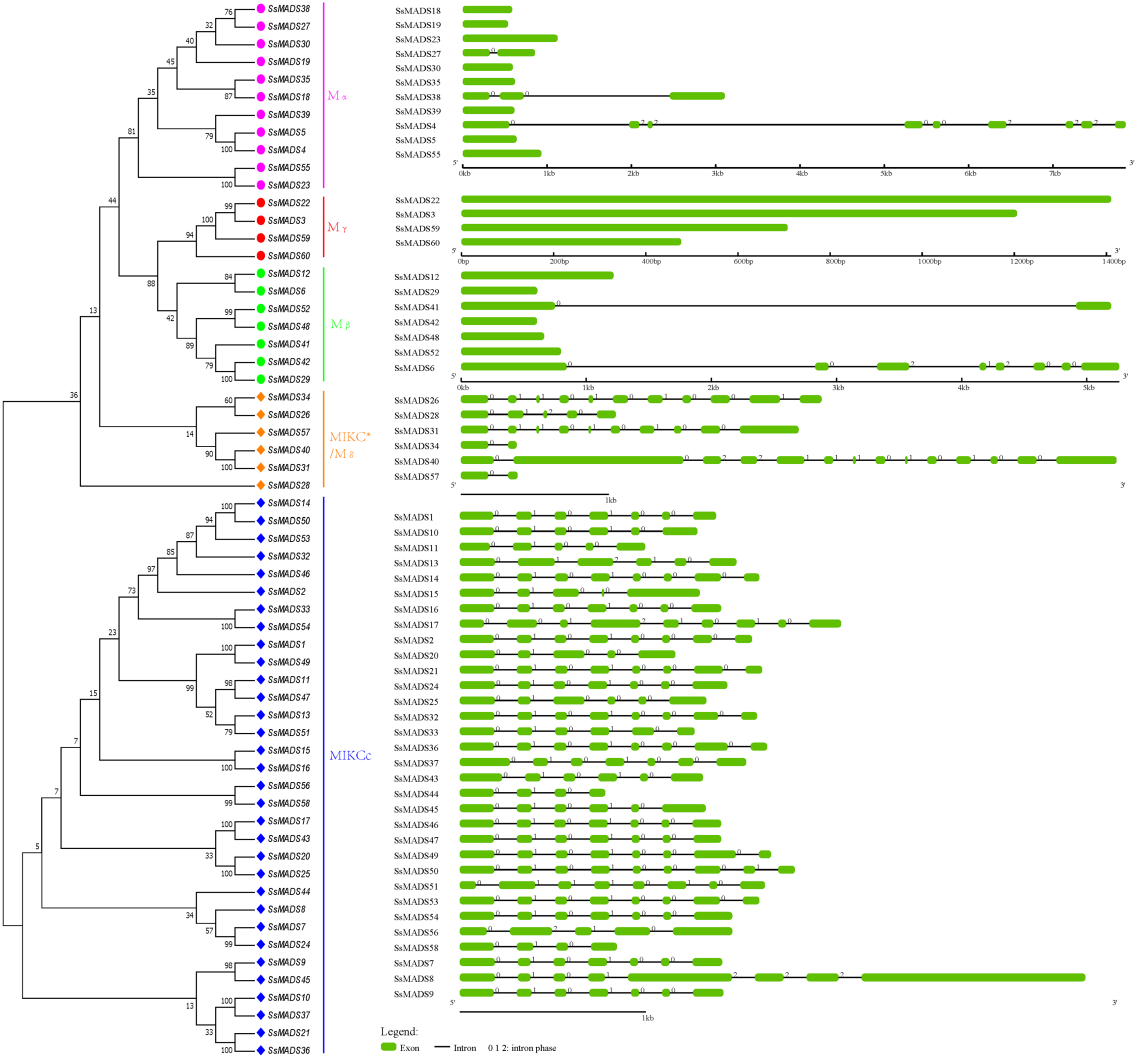
Phylogenetic relationships and gene structures of the willow MADS-box genes. An unrooted NJ tree was constructed based on the full-length willow MADS-box protein sequences. The exon-intron structures of the willow MADS-box genes were displayed using the online tool GSDS.

To gain insights into the structural diversity of willow MADS-box genes, we analyzed the exon-intron organization of the coding sequences of each willow MADS-box gene. A striking bimodal distribution of introns was observed in the *Arabidopsis*, cucumber and apple MADS-box family genes; the MIKCc and MIKC*(M*δ*) genes contained multiple introns, whereas the Mα, Mβ, and Mγ genes usually had either no or a single intron (Hu & Liu, 2012; Parenicova et al., 2003; Tian et al., 2015). We found a similar finding in willow. In Fig. 4, the SsMADS gene phylogenetic tree and the corresponding exon-intron structures are shown in the left and right panels, respectively. Among the 38 MIKC-type members, 34 (89%) members contained at least four introns, and the maximum of 13 introns was detected in *SsMADS40*. Correspondingly, among the 22 M-type genes, most of the members had no intron (77%) or a single intron, especially the Mγ-type SsMADS genes, and none of these four genes had any introns. Regardless, we found seven introns in *SsMADS6* and eight introns in *SsMADS8*.

The following interesting phenomenon was also observed: the number of introns in the six MIKC*-type willow MADS-box genes was quite varied. Among these genes, *SsMADS40* contained 13 introns, *SsMAADS26* contained 10 introns, *SsMADS31* contained nine introns, *SsMADS28* contained four introns, and SsMADS34 and *SsMADS56* contained only one intron each.

### Gene duplication events and conserved motifs in willows

Two or more adjacent homologous genes located on a single chromosome are considered tandem duplication events (TDs), whereas homologous gene pairs between different chromosomes are defined as segmental duplication events (SDs) (Bi et al., 2016; Liu & Ekramoddoullah, 2009). In this study, we identified a total of 12 homologous gene pair (including 24 SsMADS genes) duplication events. Among them, 20 genes were MIKC-type genes (18 MIKCc and two MIKC*), and the remaining four genes were classified as Mα (Table S2). Besides, among the 12 homologous gene pairs, two appeared to have undergone TDs, and ten participated in SDs.

**Figure 5:**
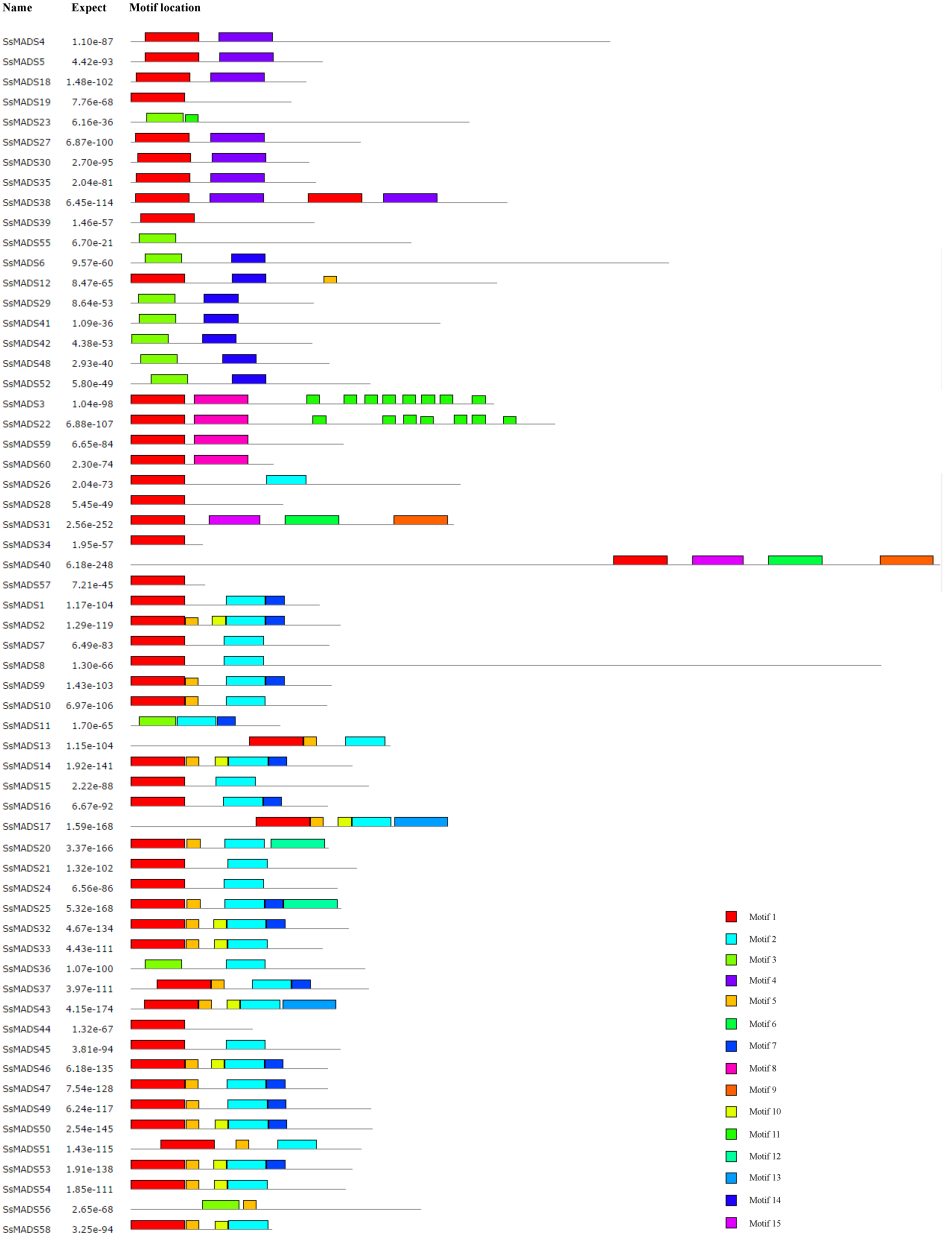
Conversed motif distributions of the willow MADS-box proteins. A total of 15 conserved motifs of the 60 willow MADS-box proteins were identified using MEME. Motifs 1–15 are indicated by different colours.

The conserved motifs of the 60 MADS-box proteins were predicted by the MEME programme to better analyze the sequence characteristics and structural differences among these genes. A total of 15 conservative motifs were predicted, and named from Motif 1 to Motif 15 (Fig. 5, Table S3).

Among these, Motif 1 and Motif 3 were widely present in all SsMADS genes. These two motifs were MADS domains, and Motif 1 was the most typical MADS domain. Motif 2 was a highly conserved K domain motif that is essential for protein interactions between MADS-box transcription factors and was present in all MIKC-type SsMADS genes except *SsMADS44* and *SsMADS56*. Interestingly, the K-box domain was identified in *SsMADS44* using the SMART programme but was not found using MEME because the two programmes used different algorithms. Further observation revealed that the K-box domain of *SsMADS44* consisted of only 53 amino acids, whereas most K-box domains in willows were 92–93 amino acids in length; this shorter length might have been due to loss of a portion of the gene during evolution, which resulted in its distinctive features. Overall, SsMADS genes of the same subgroup had similar motifs, and we speculated that they might have similar functions. A total of six basic leucine zipper (bZIP) motifs were found in five SsMADS (*SsMADS9*, *SsMADS16*, *SsMADS18*, *SsMADS19*, and *SsMADS46*) using 2ZIP, and these motifs play important roles in the expression and regulation of higher plant genes. The activation domain LXXLL motif and the inhibitory domain LXLXLX motif were also found in willow MADS-box genes. In general, a large number of motifs with different structures and functions were found in the willow MADS-box gene family, indicating that the MADS-box genes play a variety of important roles in the gene regulatory network of willows.

### Expression profiles of willow MADS-box genes in different tissues

**Figure 6:**
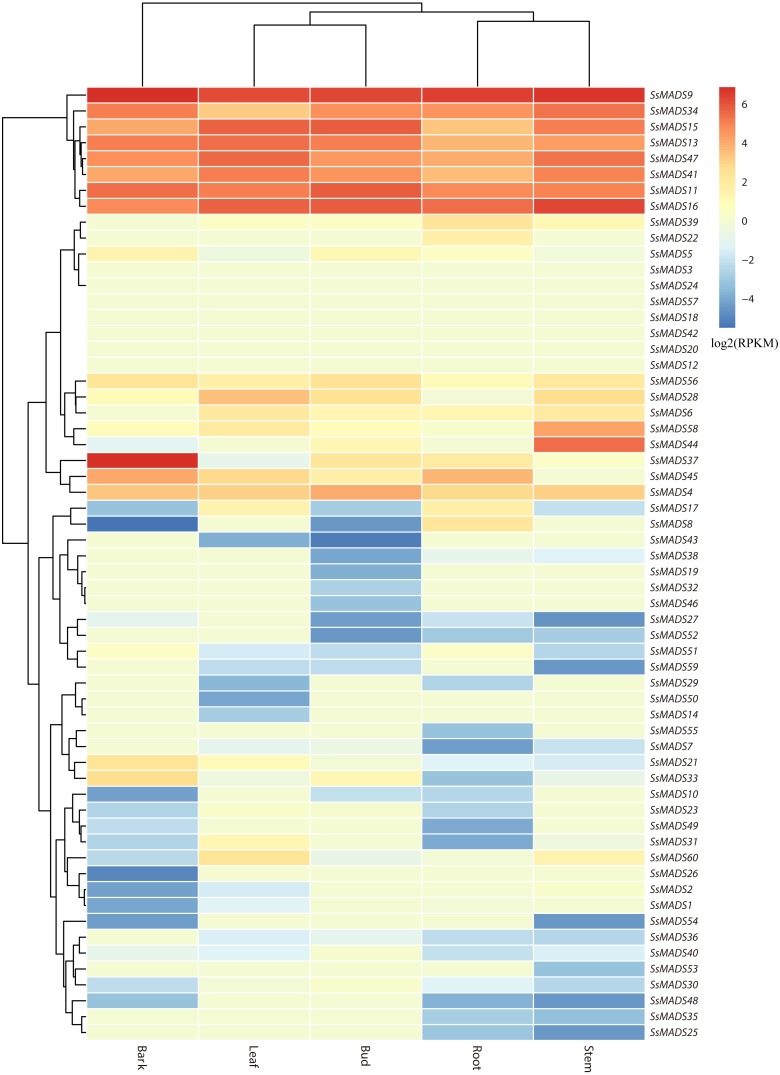
Expression analysis of the 60 willow MADS-box genes in five tissues (bark, leaf, bud, root and stem). The colour scale represents RPKM normalized log2-transformed counts. The red blocks indicate high expression, the blue blocks indicate low expression, and the light green blocks indicate no expression in this tissue.

The expression profile heat map of 60 SsMADS genes drawn using R is shown in Fig. 6. As illustrated in Fig. 6 and Table S4, most of the MADS-box genes were expressed at low levels or not expressed in these five tissues; this pattern was similar to the expression patterns of the MADS-box gene family in *Medicago truncatula*, in which seven of the genes, including *SsMADS3*, *SsMADS12*, and *SsMADS18*, were not expressed in the five tissues (Zhang et al., 2014). In contrast, 26 SsMADS genes were expressed in all tissues, and eight genes, including *SsMADS9*, *SsMADS16*, and *SsMADS23*, were highly expressed. *SsMADS9* exhibited the highest expression level in four tissues (root, stem, leaf and bud) and showed high expression in bark. The gene belonging to the highly conserved MIKCc type, which can be considered the housekeeping gene of *S. suchowensis*, participates in various growth and development processes. *SsMADS37* exhibited the highest expression in bark but quite low expression in the other four tissues. Additionally, seven of the eight genes with higher expression were of the MIKC-type; six of these were of the highly conserved MIKCc type, and the remaining gene was of the MIKC* type. Overall, the total RPKM value of the SsMADS genes was 287 in root and higher than 400 in the remaining four tissues. Therefore, the expression of the SsMADS genes in root was significantly lower than that in the stem, leaves, buds and bark. Thus, the MADS-box gene family plays a major role in willow morphogenesis.

**Figure 7:**
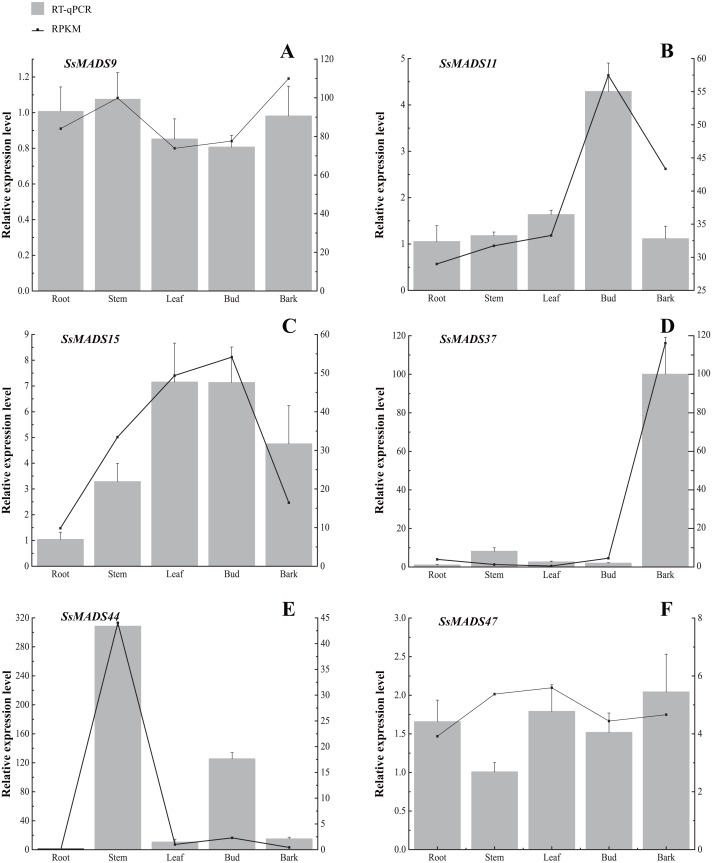
Expression patterns of 6 MIKC genes in five tissues (root, stem, leaf , bud and bark) by RT-qPCR. *SsOTU* primers were used as the internal standard for each gene. The mean expression value was calculated from 3 independent replicates. The vertical bars indicate the standard deviation. (A) *SsMADS9;* (B) *SsMADS11;* (C) *SsMADS15;* (D) *SsMADS* 37; (E) *SsMADS44*; (F) *SsMADS47*.

RT-qPCR of six MIKC-type SsMADS genes were performed to validate their expression in RNA-seq. The results suggested that the expression levels of these genes were basically consistent with that of transcriptome sequencing data (Fig. 7). Furthermore, we found an interesting gene, *SsMADS44*, which was highly expressed in the stem but expressed at extremely low levels or not expressed (root) in the other four tissues by transcriptome data. However, it was also found to be highly expressed in Bud in RT-qPCR. The reason for these divergences might be the difference in sample growth (Meng et al., 2019).

## Discussion

Systematic identification and analysis of MADS-box genes has been performed in a variety of plants, such as *A. thaliana*, rice, popular and others, but there is no large-scale study of MADS-box genes in *S. suchowensis*. In this study, we identified 60 non-redundant MADS-box genes in *S. suchowensis* and analyzed their chromosomal locations, exon-intron structures, evolution and gene expression profiles. A comparison of previous studies found that the number of MADS-box genes varies among different species. For example, 107, 105, 106 and 80 MADS-box genes were identified in *Arabidopsis thaliana*, *Populus trichocarpa*, *Giycine max* and *Prunus mume*, respectively (Leseberg et al., 2006; Parenicova et al., 2003; Shu et al., 2013; Xu et al., 2014). Surprisingly, we noticed that the number of MADS-box genes in willow and poplar was significantly different, these genes may play a role in the divergence of Salicaceae, which needs further research. In addition, it has been reported that willow might have lost more genes than poplar after the common genome duplication, which may also be the reason why the number of willow MADS-box genes is significantly less than the poplar (Dai et al., 2014). The structures of the two type MADS-box genes of *S. suchowensis* were quite different, and the type II MADS-box genes of *S. suchowensis*, particularly the MIKCc subgroup, were more conserved, which indicated that the MIKCc genes might have been subjected to greater selection pressure during evolution and are more important for the environmental adaptability of plants. Similar results were found in *Arabidopsis*, poplar, apple, wheat, soybean and *P. mume* (Leseberg et al., 2006; Parenicova et al., 2003; Schilling et al., 2019; Shu et al., 2013; Tian et al., 2015; Xu et al., 2014).

Fifty-six of the 60 SsMADS genes were distributed on 19 chromosomes. Comparing the distribution of the two major categories of MADS-box genes on willow chromosome, it was found that the MIKC-type genes are distributed across 15 of 17 willow chromosomes, whereas the M-type genes are primarily located on chromosomes 1, 4, 6 and 10. These four chromosomes account for approximately 23.05% of willow genome and contain 13.16% of the MIKC-type genes, whereas these chromosomes contain 50.00% of M-type genes. Similar results were found in soybean and apple (Shu et al., 2013; Tian et al., 2015). A total of 21 SsMADS genes in willows were clustered into 11 clusters, we hypothesized that these clustered genes play more important roles in the growth and development of willows; as a result, the clustered distribution of these genes might have given them a selective advantage during evolution, and selection could have maintained the existence of the gene clusters. For example, clustered genes co-expressed in yeast maintain a good co-expression relationship in nematodes (Hurst, Williams & Pal, 2002). However, the chromosomal distribution of the gene clusters was irregular. Related studies have suggested that the exact position and orientation of these clustered genes are not well conserved (Lee & Sonnhammer, 2003).

Different classification methods may result in different subclasses of MADS-box genes. According to the method described in *Prunus mume*, *Oryza sativa*, *Populus trichocarpa* and many other plants, the willow MADS-box genes were classified into two main groups (M-type and MIKC-type), with three subgroups in M-type (Mα, Mβ and Mγ) and two in MIKC-type (MIKCc and MIKC*) (Arora et al., 2007; Leseberg et al., 2006; Xu et al., 2014). During the classification and evolution analysis, we found that *SsMADS56* did not contain a K domain but was divided into the MIKCc subgroup and clustered with *SsMADS58*. Further research found that although this gene did not have a K domain, it contained an FMO-like domain that interfered with the formation of the K domain, probably because it had mutated during evolution. Similar phenomena have occurred in other species, such as *P. patens* (Henschel et al., 2002). After the evolution analysis, we found that the MIKC* (M*δ*) class was a transition subgroup for the type I and type II willow MADS-box genes. As shown in the phylogenetic trees described in ‘Chromosome Distribution Characteristics of the Willow MADS-box Genes’, these genes were clustered between the type I and type II genes: most of them were classified as type I, but some were categorized as type II, which might be due to the more recent emergence of type I genes compared with type II genes. The MIKC*(M*δ*) class represented a transition from type II to type I during evolution that had characteristics of the two types of SsMADS genes. This phenomenon has also been found in cucumbers, poplars and other species (Hu & Liu, 2012; Leseberg et al., 2006). Compared with those in poplar, the MIKC*(M*δ*) genes in willows were almost completely clustered in the type I cluster, which suggested that the evolution rate of willows was faster than that of poplars. In addition, by comparing the number of willow MADS-box genes with *Ginkgo biloba*, and analyzing some other gymnosperms and angiosperms, we found that although the gymnosperm genome was larger, the number of this gene family was much smaller than that of the angiosperms. We speculate that this phenomenon occurred because the MADS-box gene family mainly acts on the growth and development of flower organs, and gymnosperms generally have no obvious flowers. In contrast, angiosperms, which are also called flowering plants, have a wide variety of flowers. Therefore, the number of MADS-box genes in gymnosperms was significantly smaller than that in angiosperms.

During the analysis of orthologues genes, we found the imbalance between M-type and MIKC-type SsMADS genes, so we concluded that the MIKC-type appeared earlier than the M-type and was more conserved, whereas the M-type occurred later and evolved faster. By finding that the vast majority of SsMADS genes that did not have orthologous genes in Arabidopsis also had no orthologues genes in rice, we hypothesized that these genes might have formed after species differentiation, had unique genetic characteristics of Salicaceae plants, and might even be specific to Salicaceae plants, although these speculations require further research. The clustering of orthologous genes emphasizes the conservation and divergence of gene families, and they may contain the same functions. Specifically, the clustering of orthologous genes suggests that they might have the same or similar functions (Gabaldon et al., 2009; Ling et al., 2011; Sonnhammer & Koonin, 2002). Because most of the *Arabidopsis* MADS-box genes had functional annotations, the functions of the willow MADS-box genes could be predicted based on the orthologous gene pairs between willows and *Arabidopsis*. Functional information for the *Arabidopsis* MADS-box genes was obtained from the TAIR website. For example, the main function of the *AGL2* gene in *A. thaliana* is to regulate the development of flowers and ovules, and because *SsMADS14/32/50/53* are orthologous to this gene, it can be speculated these four genes in willow might have similar functions. *SsMADS17* and *SsMADS43* are homologous to the *Arabidopsis AGAMOUS* gene, which has a primary function of specifying the floral meristem and binding to the CArG-box sequence. The functions of other genes can be speculated in the same manner.

The exon-intron structures of multiple gene families play crucial roles during plant evolution (Bi et al., 2016). A striking bimodal distribution of introns that observed in many plants’ MADS-box family genes has also been found in willow (Hu & Liu, 2012; Parenicova et al., 2003; Tian et al., 2015). And an interesting phenomenon was also observed: the number of introns in six MIKC*-type SsMADS genes was quite varied. This dramatic change in the number of introns indicated that they were acquired or lost during evolution of the MIKC*-type willow MADS-box genes. The intron numbers of the MIKCc-type SsMADS genes were relatively stable, and further analysis showed that the intron positions of the MIKCc-type SsMADS genes were also highly conserved; this phenomenon also occurred in cucumbers, probably because these genes were purified during evolution and were more stable against environmental stress (Hu & Liu, 2012).

Gene duplication events have always been considered vital sources of biological evolution (Chothia et al., 2003). It has been proposed that gene duplications have an important role not only in genomic rearrangement and expansion but also in the diversification of gene function (Su et al., 2013; Zhang et al., 2013). According to previous studies, gene duplication caused the expansion of some willow gene families, for example, WRKY, SPL, sHsp and Hsfs (Bi et al., 2016; Feng et al., 2017; Li et al., 2018; Zhang et al., 2015). In this study, 12 homologous gene pair duplication events have been identified, including twenty MIKC-type (18 MIKCc and 2 MIKC*) and four M-type SsMADS genes, which suggested that the functions of the MIKC-type, particularly the MIKCc type, were strengthened and played more important roles in willow evolution. Almost 83.33% homologous gene pairs participated in SDs while only 16.67% participated in TDs, implying that the expression of the MADS-box gene family in willows was affected by both tandem and segmental duplication events. In contrast, the effect of SD events was greater than that of TDs, which might be due to genome-wide duplication.

In the MADS-box gene family, different subfamilies displayed different expression profiles in various species, such as *Arabidopsis*, *Medicago truncatula* and poplar (Leseberg et al., 2006; Parenicova et al., 2003; Zhang et al., 2014). For example, previous studies suggested that most M type MADS-box genes were expressed at low level or not expressed in plant tissues (Tian et al., 2015; Wei et al., 2014; Zhang et al., 2017). In the present study, the expression of willow MADS-box genes in five different tissues was analyzed using RNA-Seq data and validated by RT-qPCR. Eight SsMADS genes were found highly expressed in all five tissues, especially *SsMADS9*, which exhibited the highest expression level in four tissues (root, stem, leaf and bud) and showed high expression in bark that can be considered as the housekeeping gene of *S. suchowensis*. Except for six genes including *SsMADS4*, *SsMADS6*, *SsMADS39*, and *SsMADS41*, which are highly expressed in stem, leave, and bud, most M-type MADS-box genes are expressed weakly in willow and their function remains unclear, this pattern has also been reported in many other species such as *Arabidopsis* and sesame (Masiero et al., 2011; Wei et al., 2015). We could infer that compared with the M-type SsMADS, the MIKC-type SsMADS play more important roles in willow growth and morphogenesis. MADS-box genes in willow may have become greatly diverse and perform various functions in different tissues, the expression profiles of the MADS-box genes obtained in our study will contribute to further studies of the regulation of MADS-box genes in plant growth.

## Conclusions

Based on the latest *S. suchowensis* genome sequence and RNA-Seq data, we identified 60 SsMADS genes using bioinformatics methods and classified them as M-type (Mα, Mβ, and Mγ) and MIKC-type (MIKC*(M*δ*) and MIKCc) according to their evolutionary relationships and protein structure characteristics. We found that the gene structures of these two types were quite different, which was consistent with the results of previous research in other species. Further bioinformatics analyses performed for the obtained gene family members showed that the MIKC* (M*δ*) subclass was a transitional class between the M and MIKC types. A comparison of the numbers of MADS-box genes in gymnosperms and angiosperms showed that the numbers of genes in gymnosperms was significantly lower than that in angiosperms, further illustrating that these genes are important for the development of floral organs. In addition, after analyzing the gene structures, gene duplication events and motifs of *S. suchowensis*, we found that the MIKC type was more conserved than the M type and plays a more important role in the growth and development of *S. suchowensis*. The above results were confirmed by expression analysis of the MADS-box genes in different *S. suchowensis* tissues. In summary, the results of this study establish a foundation for a better comprehensive identification of MADS-box genes in *S. suchowensis* and a better understanding of the structure-function relationship between SsMADS genes. Compared with the related genera of poplar, which is the model species of woody plants, willow has a shorter generation period and a higher evolutionary rate and is thus easier to study (Dai et al., 2014). Our study of the willow MADS-box gene family might also provide a useful genetic database for molecular analyses of woody plants.

##  Supplemental Information

10.7717/peerj.8019/supp-1 Table S1 The primer information of SsMADS genes used in this study

10.7717/peerj.8019/supp-2 Table S2 Homologous genes in the willow MADS-box gene family

10.7717/peerj.8019/supp-3 Table S3 The details of fifteen conserved motif sequences identified in SsMADS genes

10.7717/peerj.8019/supp-4 Table S4 The details of total transcript abundance of SsMADS genes by RPKM annotation

10.7717/peerj.8019/supp-5 Figure S1 Sequence logo of the 60 willow MADS-box domains The logo was generated using the web-based application WebLogo3 (http://weblogo.threeplusone.com) with the default parameters. The heights of the symbols within each stack indicate the relative frequency of each amino acid at that position.

10.7717/peerj.8019/supp-6 Figure S2 Phylogenetic tree of *S. suchowensis* and *O. sativa* MADS-box domains A total of 60 MADS-box domains from S. suchowensis and 66 from O. sativa were used to construct a NJ tree using MEGA 7. Different shapes and colours represent different species and gene categories.

10.7717/peerj.8019/supp-7 Figure S3 Phylogenetic tree of *S. suchowensis* and *P. trichocarpa* MADS-box domains A total of 60 MADS-box domains from S. suchowensis and 103 from *P. trichocarpa* were used to construct a NJ tree using MEGA 7. Different shapes and colours represent different species and gene categories.

## Additional Information and Declarations

### Data Availability

The following information was supplied regarding data availability: The latest *S. suchowensis* genome, annotation information, coding sequences and protein sequences are available at Figshare: Qu, Yanshu; Bi, Changwei; He, Bing; Ye, Ning; Yin, Tongming; Xu, Li-an (2019): Willow gene family. figshare. Dataset. https://doi.org/10.6084/m9.figshare.9878582.v1. They’re also available at the laboratory website: http://bio.njfu.edu.cn/ss_wrky.
