# Supplementary material for: Genome-wide identification and characterization of the MADS-box gene family in Salix suchowensis
Source: PeerJ. 2019 Nov 7;7:e8019. doi: 10.7717/peerj.8019 (PMC6842560; doi:10.7717/peerj.8019)
Supplement: Table S1 [file peerj-07-8019-s001.pdf]

	Forward primers	Reverse primers
SsMADS9	5′ TGCTACCGGCAAGCTCTTCG 3′	5′ CTGCAACTCAAGAGACGGCTGA 3′
SsMADS11	5′ AGCCATGGCAAGTCGCAAGA 3′	5′ GGCGCTTGGTTCTTGTTTCCG 3′
SsMADS15	5′ CCAAGAGGCGACATGGACTGT 3′	5′ AGATGAGGAGGGCGATTTGCAC 3′
SsMADS37	5′ AAACCCCAAGATTAGCAGGA 3′	5′ CTCCGATTTCCGCATCACAC 3′
SsMADS44	5′ TGAGCCACAGACTAGATCCTT 3′	5′ CGCTCGCTCTAGTTGCTT 3′
SsMADS47	5′ TGGCCAGTCTCAAGGGAACAGA 3′	5′ TCTTGGAGGGAAGCGCTTTGT 3′
SsOTU	5′ TGGAGCCCTGCCCTTACCAT 3′	5′ TGTGCGCAATCTCAGGCACT 3′
