# Supplementary material for: Genome-wide identification and characterization of the MADS-box gene family in Salix suchowensis
Source: PeerJ. 2019 Nov 7;7:e8019. doi: 10.7717/peerj.8019 (PMC6842560; doi:10.7717/peerj.8019)
Supplement: Table S2 [file peerj-07-8019-s002.pdf]

No.	Gene name	Homologous gene	Category	Identify (%)
1	SsMADS1	SsMADS49	MIKCc	74.27
2	SsMADS4	SsMADS5	Mα	96.26
3	SsMADS11	SsMADS47	MIKCc	66.51
4	SsMADS13	SsMADS51	MIKCc	86.49
5	SsMADS14	SsMADS50	MIKCc	91.29
6	SsMADS15	SsMADS16	MIKCc	81.82
7	SsMADS17	SsMADS43	MIKCc	88.99
8	SsMADS20	SsMADS25	MIKCc	79.83
9	SsMADS27	SsMADS38	Mα	65.49
10	SsMADS31	SsMADS40	MIKC*(Mδ)	86.51
11	SsMADS32	SsMADS53	MIKCc	67.90
12	SsMADS56	SsMADS58	MIKCc	80.20
