# Supplementary material for: Genome-wide identification and characterization of the MADS-box gene family in Salix suchowensis
Source: PeerJ. 2019 Nov 7;7:e8019. doi: 10.7717/peerj.8019 (PMC6842560; doi:10.7717/peerj.8019)
Supplement: Table S3 [file peerj-07-8019-s003.pdf]

| Motif | Width | Best possible match |
|---|---|---|
| 1 | 50 | KRIENKTSRQVTFSKRRNGLLKKAYELSVLCDAEVALIIFSPTGKLYEFS |
| 2 | 50 | EAAKLKKKIEKLQRSQRNLLGEDLDPLSIKELQQLEKQLERSLKRIRARK |
| 3 | 8 | MGRGKVZJ |
| 4 | 41 | RKKSFRKRKAGLLKKASELAILCGVEACVIIFGPDGTEETW |
| 5 | 15 | SSSMEKTJERYQKCS |
| 6 | 29 | TQLLLEQJEDLQRKERLLLEENAALKEKJ |
| 7 | 50 | IEAHRNARILELNQELTZILKQLEAEKKRGEELDQMRKANGSZYWWEAPI |
| 8 | 50 | FQNJPAMERSKKMVNQESYLKERIGKLNDQLRKQRKDNRELEMEDLMQQV |
| 9 | 50 | HKENLGKQHFMSLECHGQFQNGMHVPFRMGAEQQLPPJLWIPNNDSQHIM |
| 10 | 14 | GDSAGGNNFDPWLL |
| 11 | 50 | AAEMKLQKGPEDFNVGGSFEAPKPEYDSGPCGWASAPGSCAVTMFDDHLY |
| 12 | 49 | ENERKRQNMNLMPGGVEFEIMQPQPFDSRNYSQVNGLPPANHYPHDDQM |
| 13 | 47 | PVEHQQVLDQFPFCGEPSSVLQLSNISHQIDPYHLQLAQPSLQGSNV |
| 14 | 38 | AEAKFETWDDRLNYLPEESLRDLLKILERKLQVVDEKV |
| 15 | 50 | TFKKLDHDVNIPEFLGSSSQSIEDLTSQSRLLQNQLSDVHKRLSFWTNPD |
