# Supplementary material for: Genome-wide identification and characterization of the MADS-box gene family in Salix suchowensis
Source: PeerJ. 2019 Nov 7;7:e8019. doi: 10.7717/peerj.8019 (PMC6842560; doi:10.7717/peerj.8019)
Supplement: Table S4 [file peerj-07-8019-s004.pdf]

Name	Root	Stem	Leaf	Bud	Bark
SsMADS1	0.945761991	0	0.430158941	0	0.05804
SsMADS2	0	1.234926703	0.317206554	0	0.052311
SsMADS3	0	0	0	0	0
SsMADS4	6.903528207	8.532136289	8.877137264	15.18168679	10.20287
SsMADS5	1.438623874	0.859652349	0.693982415	2.0994325	2.660837
SsMADS6	2.408836749	3.818364735	4.326522357	2.384516788	0.991837
SsMADS7	0.051353592	0.260396309	0.520225128	0.665238885	0
SsMADS8	4.833641405	0	0	0.044282468	0.022027
SsMADS9	84.02437923	99.89303475	73.8354758	77.601881	109.9138
SsMADS10	0.182211026	0	0	0.22479785	0.05591
SsMADS11	28.98817476	31.73626367	33.29373666	57.4565022	43.35676
SsMADS12	0	0	0	0	0
SsMADS13	13.43770162	19.89097376	43.31325777	34.94650391	32.26951
SsMADS14	0	0	0.133530492	0	0
SsMADS15	9.844522544	33.43070759	49.36607104	54.13391929	16.45889
SsMADS16	45.24110785	75.72679222	50.09771379	57.78895771	27.07595
SsMADS17	3.265708072	0.238477428	2.901149657	0.139618038	0.104174
SsMADS18	0	0	0	0	0
SsMADS19	0	0	0	0.068444038	0
SsMADS20	0	0	0	0	0
SsMADS21	0.384333558	0.333488356	1.995775457	0.878592392	5.171552
SsMADS22	3.163272021	0	0	0	0
SsMADS23	0.16645411	0.962614139	1.226344036	1.110801108	0.162511
SsMADS24	0	0	0	0	0
SsMADS25	0.121251537	0.044714518	0	0	0
SsMADS26	0	0	0	0	0.033393
SsMADS27	0.244785457	0.041032146	0	0.048045031	0.501873
SsMADS28	0.934635379	6.647207607	11.158829	5.332998398	1.971645
SsMADS29	0.167721338	0	0.080907638	0	0
SsMADS30	0.40123236	0.184955505	0.91245836	1.206585428	0.215451
SsMADS31	0.063403039	0.701443385	2.339767526	1.009549561	0.170228
SsMADS32	0	0	0	0.151877886	0
SsMADS33	0.106564731	0.589475896	0.732536993	2.444544691	6.551955
SsMADS34	23.88924733	39.91515953	9.023199337	26.54487941	34.98509
SsMADS35	0.137732329	0.101584438	0	0	0
SsMADS36	0.218252767	0.181093797	0.347436068	0.518331965	0
SsMADS37	3.95500469	1.307899645	0.528811095	4.571102486	116.1356
SsMADS38	0.544323448	0.376373999	0	0.058760109	0
SsMADS39	5.201690951	2.205477832	1.449198572	1.351266486	0.985823
SsMADS40	0.234591242	0.362412416	0.385373475	1.129326627	0.544731
SsMADS41	12.09362126	30.09313119	32.63302245	23.68143761	16.89668
SsMADS42	0	0	0	0	0
SsMADS43	0	0	0.072036186	0.026867287	0

SsMADS44	0	44.04544392	1.026515655	2.297153026	0.448101
SsMADS45	13.6141018	0.987941363	7.366241094	3.128597541	16.24242
SsMADS46	0	0	0	0.111885688	0.973955
SsMADS47	15.10628057	41.44668281	48.26029764	21.73379484	25.295
SsMADS48	0.077030388	0.047344784	0	0	0.110302
SsMADS49	0.063759235	0	0	0	0.205422
SsMADS50	0	0	0.061284517	0	0
SsMADS51	1.307811503	0.183923388	0.320786142	0.215358096	1.380712
SsMADS52	0.127997863	0.137673647	0	0.046058206	0
SsMADS53	0	0.106333304	0	0	0
SsMADS54	0	0.043779068	0	0	0.050997
SsMADS55	0.109477272	0	0	0	0
SsMADS56	1.938518367	4.354249749	2.932901871	5.650140362	4.882911
SsMADS57	0	0	0	0	0
SsMADS58	1.156600652	18.59383444	3.870683234	1.794803214	1.863188
SsMADS59	0	0.044335581	0.208782845	0.207652251	0
SsMADS60	0.892228294	2.53354145	5.216507227	0.577900132	0.191641
