# Supplementary figures and images for: Genome-wide identification and characterization of the MADS-box gene family in Salix suchowensis
Source: PeerJ. 2019 Nov 7;7:e8019. doi: 10.7717/peerj.8019 (PMC6842560; doi:10.7717/peerj.8019)

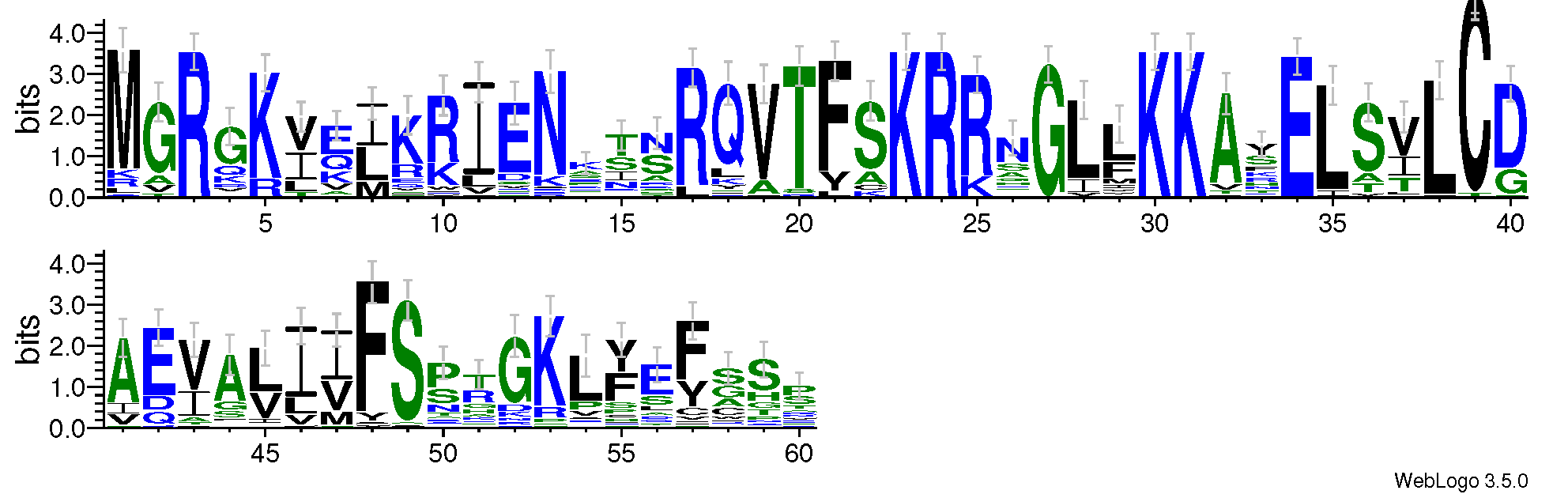

Supplement: Figure S1 — The logo was generated using the web-based application WebLogo3 (http://weblogo.threeplusone.com) with the default parameters. The heights of the symbols within each stack indicate the relative frequency of each amino acid at that position. [file peerj-07-8019-s005.png]

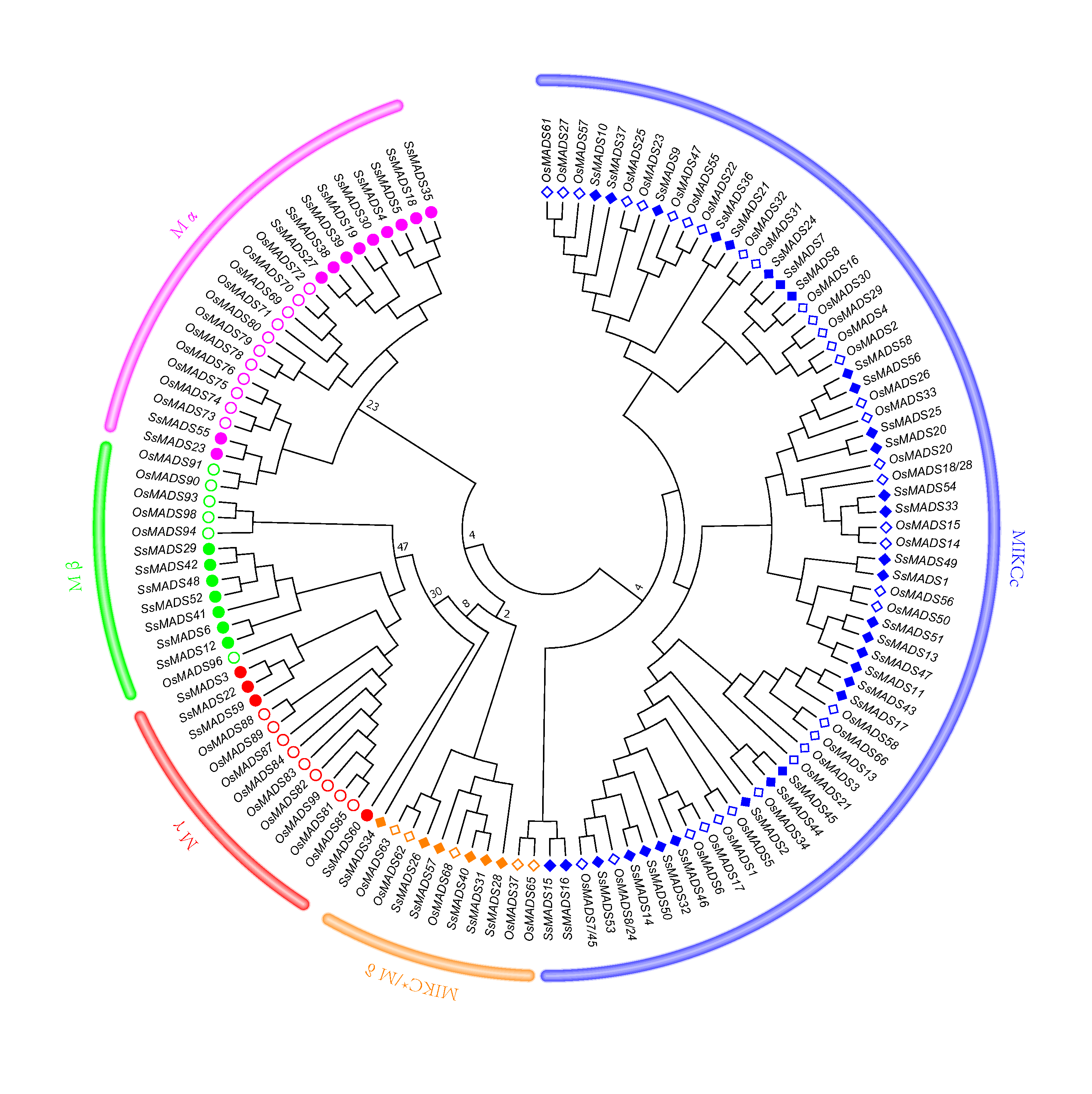

Supplement: Figure S2 — A total of 60 MADS-box domains from S. suchowensis and 66 from O. sativa were used to construct a NJ tree using MEGA 7. Different shapes and colours represent different species and gene categories. [file peerj-07-8019-s006.png]

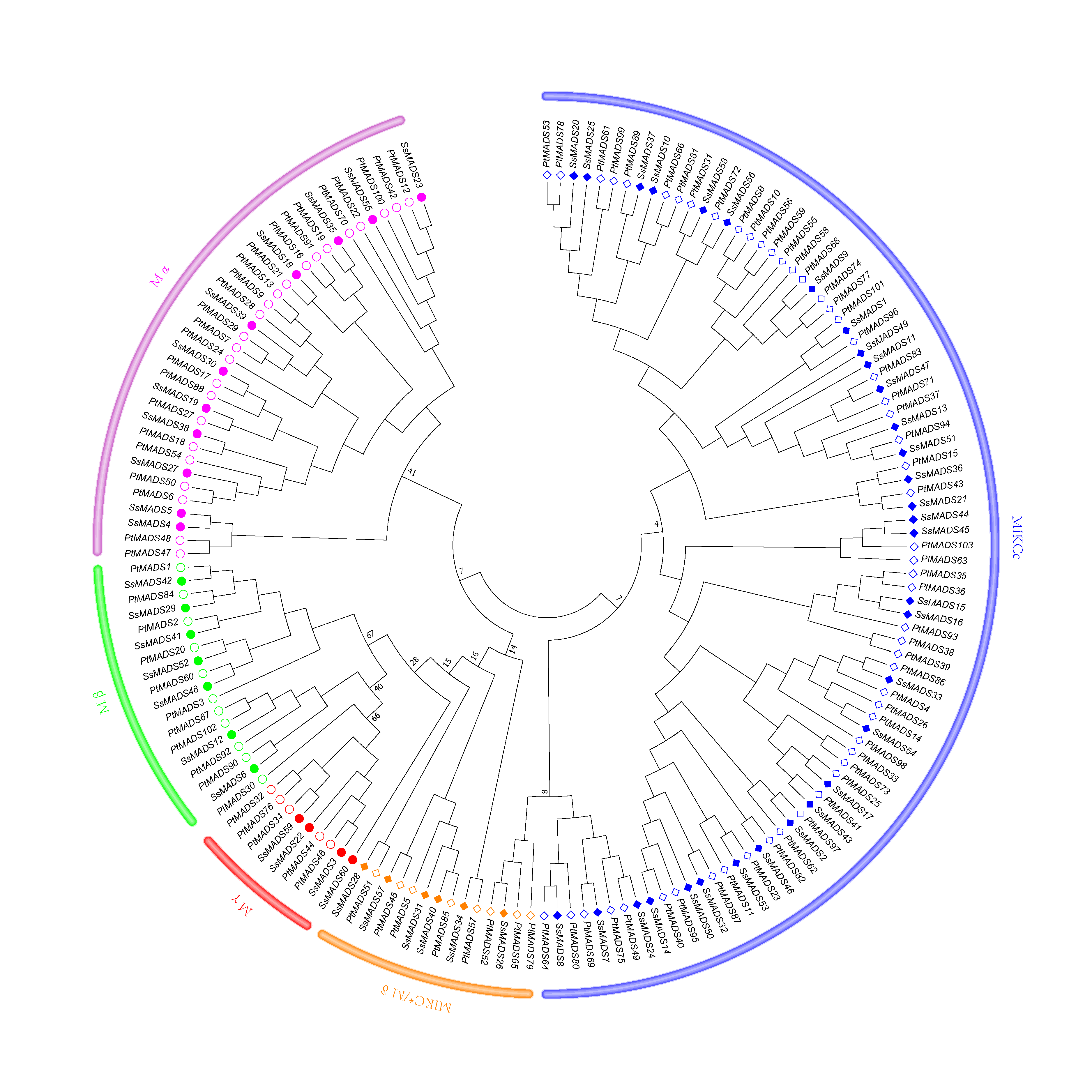

Supplement: Figure S3 — A total of 60 MADS-box domains from S. suchowensis and 103 from P. trichocarpa were used to construct a NJ tree using MEGA 7. Different shapes and colours represent different species and gene categories. [file peerj-07-8019-s007.png]
